# White matter integrity changes and neurocognitive functioning in adult-late onset DM1: a follow-up DTI study

**DOI:** 10.1038/s41598-022-07820-1

**Published:** 2022-03-07

**Authors:** Garazi Labayru, Borja Camino, Antonio Jimenez-Marin, Joana Garmendia, Jorge Villanua, Miren Zulaica, Jesus M. Cortes, Adolfo López de Munain, Andone Sistiaga

**Affiliations:** 1 Department of Clinical and Health Psychology and Research Methodology, Psychology Faculty, University of the Basque Country (UPV/EHU), Avda. Tolosa, 70. 20018 Donostia-San Sebastián, Gipuzkoa, Spain; 2 Neuroscience Area, Biodonostia Health Research Institute, Donostia-San Sebastián, Gipuzkoa, Spain; 3 Center for Biomedical Research Network (CIBER), Institute of Health Carlos III, Madrid, Spain; 4 Biocruces-Bizkaia Health Research Institute, Barakaldo, Spain; 5 Biomedical Research Doctorate Program, University of the Basque Country (UPV/EHU), Leioa, Spain; 6 Osatek, Donostia University Hospital, Donostia-San Sebastián, Gipuzkoa, Spain; 7 Department of Cell Biology and Histology, University of the Basque Country (UPV/EHU), Leioa, Spain; 8 IKERBASQUE, The Basque Foundation for Science, Bilbao, Spain; 9 Neurology Department, Donostia University Hospital, Donostia-San Sebastián, Gipuzkoa, Spain; 10 Neuroscience Department, University of the Basque Country (UPV/EHU), Donostia-San Sebastián, Gipuzkoa, Spain

**Keywords:** Cognitive ageing, Neural ageing, Neural circuits, Neurodegeneration, Neuromuscular disease

## Abstract

Myotonic Dystrophy Type 1 (DM1) is a multisystemic disease that affects gray and white matter (WM) tissues. WM changes in DM1 include increased hyperintensities and altered tract integrity distributed in a widespread manner. However, the precise temporal and spatial progression of the changes are yet undetermined. MRI data were acquired from 8 adult- and late-onset DM1 patients and 10 healthy controls (HC) at two different timepoints over 9.06 years. Fractional anisotropy (FA) and mean diffusivity (MD) variations were assessed with Tract-Based Spatial Statistics. Transversal and longitudinal intra- and intergroup analyses were conducted, along with correlation analyses with clinical and neuropsychological data. At baseline, reduced FA and increased MD values were found in patients in the uncinate, anterior-thalamic, fronto-occipital, and longitudinal tracts. At follow-up, the WM disconnection was shown to have spread from the frontal part to the rest of the tracts in the brain. Furthermore, WM lesion burden was negatively correlated with FA values, while visuo-construction and intellectual functioning were positively correlated with global and regional FA values at follow-up. DM1 patients showed a pronounced WM integrity loss over time compared to HC, with a neurodegeneration pattern that suggests a progressive anterior–posterior disconnection. The visuo-construction domain stands out as the most sensitive neuropsychological measure for WM microstructural impairment.

## Introduction

Myotonic Dystrophy Type-1 (DM1) is an inherited autosomal dominant disorder, which affects multiple systems (e.g., muscular, cardiovascular, respiratory, gastrointestinal, endocrine), including the Central Nervous System (CNS). Although considered a rare disease, with an estimated worldwide prevalence of 0.5–18.1 per 100,000 inhabitants^1^, DM1 is the most prevalent form of adult muscular dystrophy. Moreover, such prevalence is significantly higher in the geographical area of Gipuzkoa (Spain), which is considered the second most affected area in the world, with an estimated prevalence of 26.5 per 100,000 inhabitants^2^.

Regarding CNS involvement, several symptoms such as excessive daytime sleepiness, fatigue, cognitive deficits, mood and behavior disorders, and certain personality traits, have been reported in varying degrees^3^. Additionally, neuroimaging studies have described brain structural abnormalities in both grey (GM) and whiter matter (WM) tissue, as well as disrupted connectivity patterns^4–7^. Several studies have shown that these structural and connectome impairment profiles are significantly correlated with DM1 patients’ clinical features such as CTG expansion and muscular impairment^8–12^, and with cognitive functions including intellectual functioning, visuospatial/visuoconstructive skills, and executive functioning^13–16^.

According to a relatively recent review^17^, DM1 patients present general atrophy and GM volume reductions in all four cortical lobes (parietal, temporal, frontal and occipital), the basal ganglia, and the cerebellum. WM lesions (WML) and WM hyperintensities (WMH) have also been reported, as well as cortical volume loss and corpus callosum atrophy^17,18^. In addition, WM tractography studies^18,19^ have shown significant diffusivity alterations, including an increase in mean diffusivity (MD), axial diffusivity (AD), radial diffusivity (RD), and a decrease in fractional anisotropy (FA). The multiple alterations found in WM have prompted some authors to state that DM1 is a predominantly white matter disease^9^.

Although still a matter of debate, there is a neurodegenerative hypothesis concerning the progression of the disease, supported by neuropathological, neuropsychological and neuroimaging evidence^15,20–24^. However, longitudinal studies assessing precise neurodegenerative patterns are scarce, and very few have assessed WM variations over time^14,15,25,26^. Of these studies, only two have attempted to analyze diffusivity values, yielding mixed results. Researchers agree that further investigation of WM pathology should be conducted with more valid and novel techniques such as Diffusion Tensor Imaging (DTI) tractography. This technique, along with a set of neuropsychological measures, are potentially critical for fully characterizing the status of individuals with DM1^10^.

The aim of this study is to assess WM brain alterations by longitudinally analyzing diffusion images over a period of 10 years in clinical, neuropsychological and molecularly well characterized adult and late onset DM1 individuals. By comparing these individuals with healthy controls, it should be possible to delineate the pathway of disease progression and explore how these evolving patterns are associated with clinical and neuropsychological functioning.

## Results

**Figure 1:**
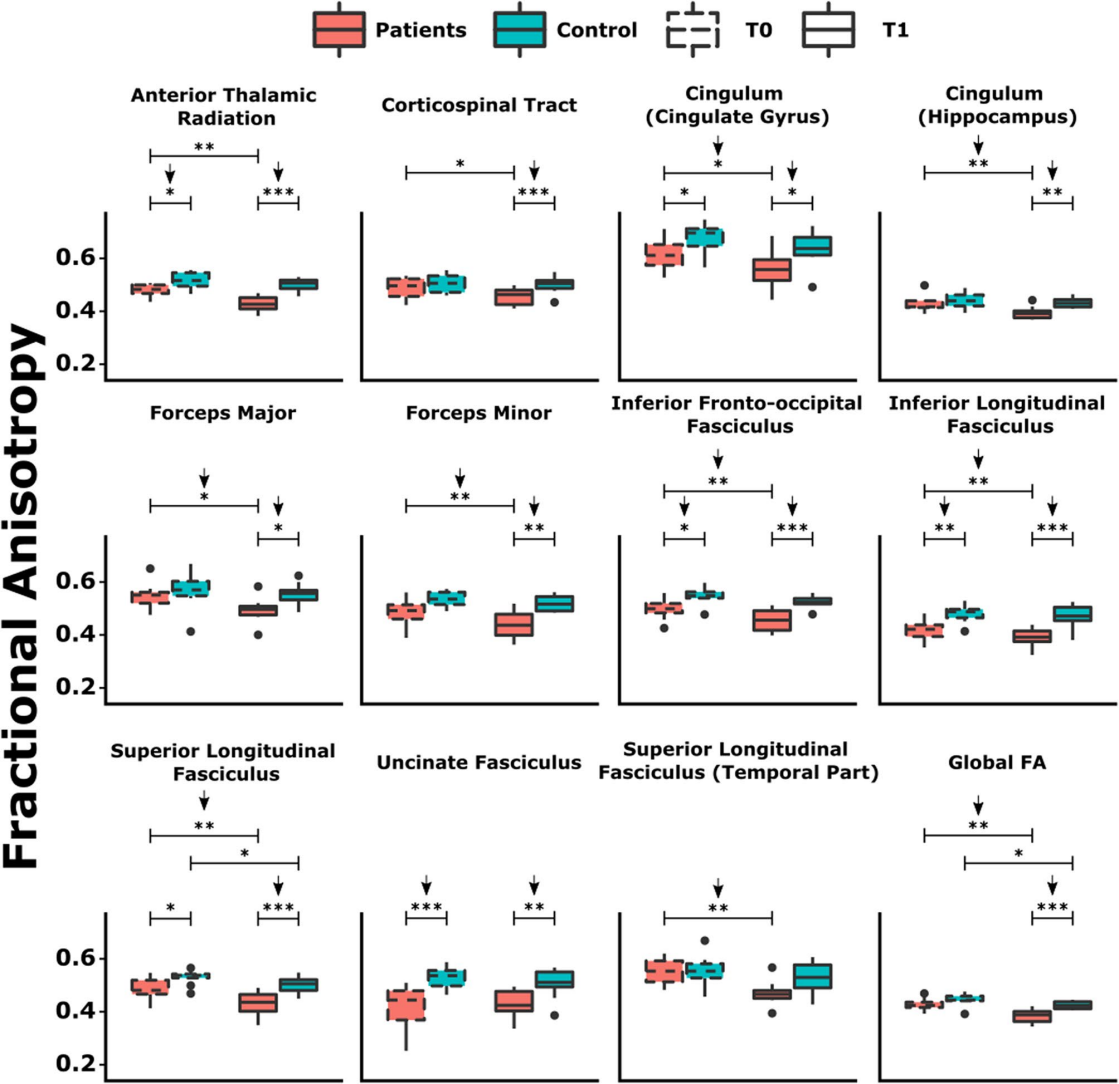
Differences in global and major tract integrity as measured by fractional anisotropy (FA) between groups and across longitudinal measures. For FA, lower values indicate reduced tract integrity. *p < 0.05; **p < 0.01; ***p < 0.005. Arrows pointing down indicate comparisons surviving FDR multiple corrections.

**Figure 2:**
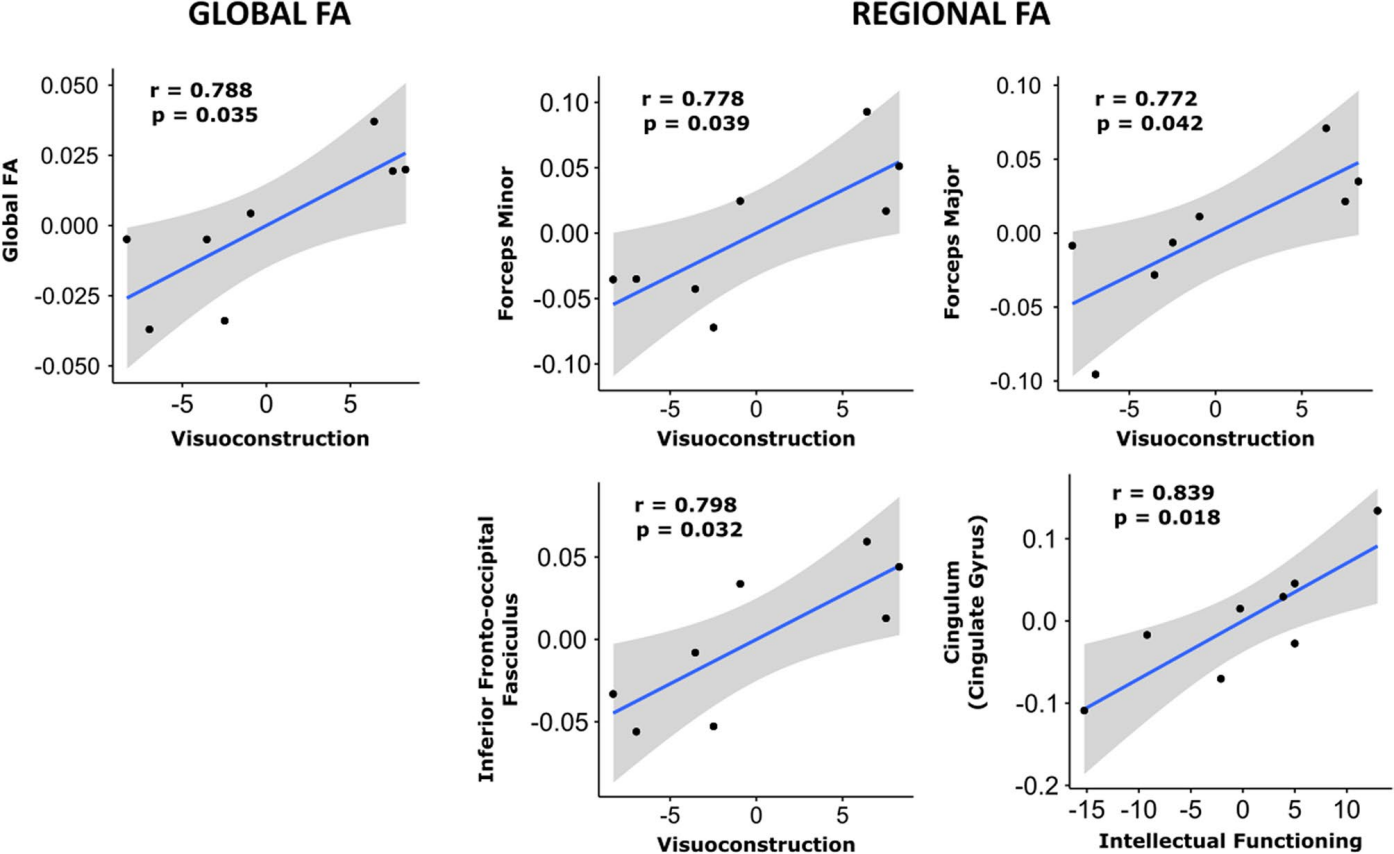
Association between tract integrity as measured by FA and neuropsychological scores. Shaded areas represent the 95% confidence interval in the regression model. Among all possible comparisons, only those plots with a statistically significant Pearson correlation r (uncorrected, *p*-value < 0.05) are displayed.

Across all participants, the mean time from baseline to follow-up was 9.06 (SD = 0.46) years, with no differences between groups (DM1: $\overline{x }$= 9.24, SD = 0.33; HC: $\overline{x }$= 8.91, SD = 0.5; *t* =  − 1.59, *p* = 0.131, *d* = 0.76). Regarding demographic characteristics of the sample, statistical analyses did not show significant differences between DM1 and HC groups in terms of gender, age, or years of education (Table 1; Figs. 1, 2).

**Table 1:** Demographic characteristics of the sample at baseline and follow-up.

		DM1 (*N* = 8)		HC (*N* = 10)		Statistic	*p*	Effect size
		Mean/*N* (%)	(SD)	Mean/*N* (%)	(SD)			
Gender	Male	4 (50%)		5 (50%)		*X*^2^ = 0.000	1	*V* = 1
	Female	4 (50%)		5 (50%)				
Age at baseline		46.5	(10.3)	44.4	(8.6)	*t* = − 0.472	0.643	*d* = 0.22
Age at follow up		55.75	(10.25)	53.3	(8.89)	*t* = − 0.543	0.595	*d* = 0.26
Years of education		14.5	(7.35)	14.13	(4.25)	*t* = − 0.125	0.902	*d* = 0.06

DM1: Myotonic Dystrophy Type 1; HC: Healthy controls; SD: Standard Deviation. Descriptive data are shown as mean and SD for age at baseline, age at follow-up and years of education. Frequency (*N*) and percentage (%) are shown for gender.

**Figure 3:**
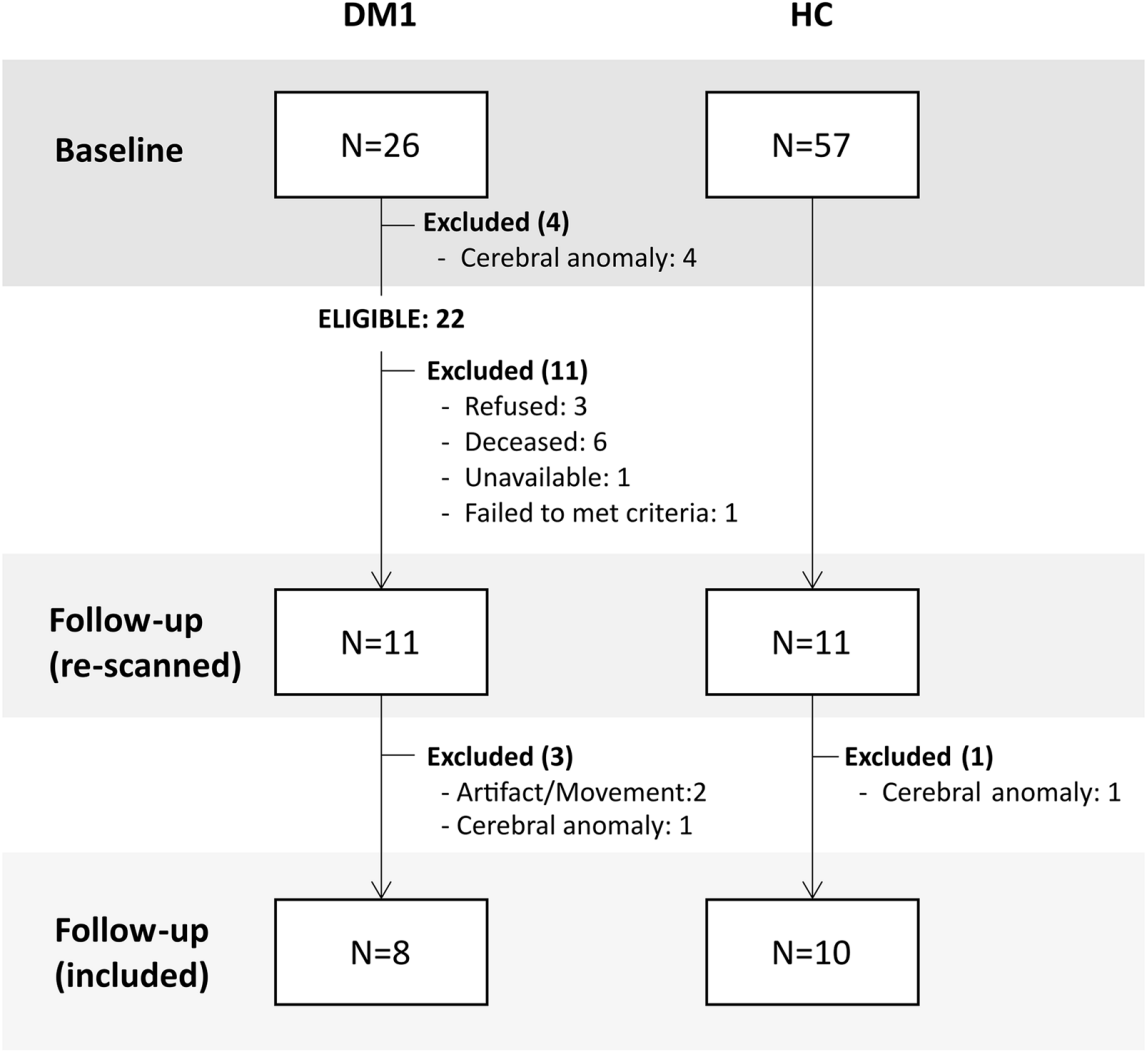
Flow-chart of the sample selection process.

From the eligible DM1 patients (*N* = 22, see Fig. 3), those who were finally included for analyses (*N* = 8) did not differ from those who were excluded (*N* = 14) in terms of age at baseline (*t* = 1.07, *p* = 0.296), muscular impairment at baseline (*U* = 14.5, *p* = 0.0.86), years of education (*U* = 36, *p* = 0.167) and estimated intellectual functioning at baseline (*t* =  − 1.42, *p* = 0.17). However, CTG expansion size at baseline was statistically larger (*t* = 2.82, *p* = 0.01) in the excluded patients ($\overline{x }$=820.29, SD = 510–27) than in the included patients ($\overline{x }$=277.25, SD = 232.23).

WM lesion quantification revealed several differences between DM1 and HC both at baseline and follow-up (Table 2). In particular, DM1 patients showed significantly greater WM lesions cortically, but not in subcortical regions. Regions with larger WM lesion load at baseline were the total brain and temporal lobe; and at follow-up parieto-occipital and temporal lobes. Intragroup analyses of WM lesion load progression revealed a statistically significant increase in DM1 patients (*z* = −2.21; *p* = 0.027), but not in HC (*z* = −1.46; *p* = 0.142). Longitudinal analysis of clinical data revealed an absence of significant worsening of genetic defect (*t* = −2.109; *p* = 0.08) and muscular impairment (*Z* = −1; *p* = 0.317).

**Table 2:** Clinical characterization of the sample at baseline and at follow-up.

	Baseline				*U*	*p*	*r*	Follow-up				*U*	*p*	*r*
	HC		DM1					HC		DM1				
	Mean	SD	Mean	SD				Mean	SD	Mean	SD			
**WM lesion load**														
GLOBAL	1.1	(0.99)	5.33	(5.98)	19.50	0.034	0.49	2.3	(2.83)	6.89	(6.31)	22.00	0.058	0.43
Frontal	0.6	(0.7)	1.89	(2.03)	27.00	0.121	0.36	1.3	(1.34)	2.44	(2.13)	31.00	0.229	0.28
Parieto-occipital	0.3	(0.48)	1.89	(2.42)	24.00	0.059	0.43	0.4	(0.7)	2.67	(2.5)	20.50	0.030	0.50
Temporal	0	(0)	1.22	(1.99)	25.00	0.022	0.53	0	(0)	1.56	(2.19)	25.00	0.022	0.53
Basal ganglia	0.1	(0.32)	0.22	(0.67)	44.00	0.878	0.04	0.5	(1.58)	0.22	(0.67)	45.00	1.000	0.00
Infratentorial	0.1	(0.32)	0.11	(0.33)	44.50	0.939	0.02	0.1	(0.32)	0	(0)	40.50	0.343	0.22
CTG			277.25	(232.23)						437.43	(385.21)			
MIRS			2.6	(0.89)						2.13	(1.25)			

HC: Healthy Controls; DM1: Myotonic Dystrophy Type 1; SD: Standard Deviation. Descriptive data are shown as mean and SD for WM lesion load, CTG and MIRS.

The Supplementary material includes intragroup comparisons of DM1 patients’ neuropsychological performance over time (Supplementary Table 1). Attention/processing speed was the only domain in which a statistically significant decrease was observed from baseline to follow-up (measured by standardized T values).

### Intergroup and intragroup tract integrity differences: transversal and longitudinal analyses

Intergroup and intragroup differences in global and major tract integrity are shown in Fig. 1. Global FA decreased significantly over time in both patients and HC, this decrease being more marked in DM1 patients. Significant differences between DM1 and HC were found only at follow up; patients showing decreased FA values. Regarding Global MD, intergroup differences were only significant at follow up (see Supplementary tables 2 and 3).

All tracts analyzed showed significant intergroup differences in integrity, with the DM1 group showing the greatest WM disintegrity in all cases (decreased FA and increased MD). Most of the differences were found at follow up. Regarding intragroup analysis, patients suffered a significant loss of integrity in all tracts, except for the *uncinate fasciculus*; whereas HC showed a significant progressive loss in the *cingulate gyrus* and *superior longitudinal fasciculus*. Group contrasts for all four possible combinations including transversal and longitudinal differences are shown in Supplementary Table 2 for FA, and in Supplementary Table 3 for MD, while the peak ROIs of the studied WM tracts showing intergroup differences at baseline and follow-up can be found in Supplementary Tables 4 and 5, respectively.

### Association between tract integrity and clinical and neuropsychological data

Correlation analyses between clinical (CTG, MIRS, WM lesion load) and neuropsychological performance and global FA at baseline did not reveal any significant correlations. At follow-up, global FA was only significantly associated with the visuo-construction domain (*r* = 0.79, *p* = 0.035) (Fig. 2) and WM lesion load (*r* = −0.76, *p* = 0.048) (Supplementary Table 6).

Regarding tract-based FA values, associations were only found at follow-up with neuropsychological performance (Fig. 2 and Supplementary Table 6) and WM lesion load (Supplementary Table 6), except for an isolated negative correlation between WM lesion load and FA in the *inferior longitudinal fasciculus* (*r* = −0.78, *p* = 0.037). In a similar manner, the visuo-construction domain was positively associated with regional FA values in the *forceps major*, *forceps minor* and *inferior fronto-occipital fasciculus*. Estimated IQ positively correlated with cingulum FA value. In all cases, better neuropsychological performance was associated with greater FA values. Greater WM lesion load correlated with lower FA values in the *inferior fronto-occipital fasciculus, inferior longitudinal fasciculus* and *superior longitudinal fasciculus*. No other significant associations were found between regional FA and other neuropsychological and clinical data.

## Discussion

The present work constitutes the most extensive longitudinal DTI study in a sample of adult and late onset DM1 patients and HC to date. Over a period of nearly a decade, this study has revealed a progression in WM integrity impairment in adult and late onset DM1 population occurring as early as from the 40’s to the 50’s. The reduction in FA found at baseline in patients in comparison with HC was greater at follow-up, where WM integrity was compromised throughout all the tracts and similar results were also seen when assessing MD alterations. This result points in the direction of the previously hypothesized neurodegenerative process and adds relevant information about the nature and early timing of this progression.

So far, only four MRI longitudinal studies including WM progression assessment^14,15,25,26^ have been published. Regarding WM lesion load, DM1 patients in this study—but not HC—showed a progression of WM lesion load over time, which supports the idea of a gradual deterioration, as suggested by some of the previous longitudinal studies^14,25^.

Regarding WM integrity, a pattern of progressive degeneration was confirmed, evolving from the anterior thalamic radiation, the *inferior fronto-occipital*, the *longitudinal* and the *uncinate fasciculi*, to a widespread involvement at follow-up. The damage shown at baseline indicates that patients suffer a WM alteration that disrupts multiple pathways, including the prefrontal-thalamic connection (anterior thalamic radiation), with decreases in volume observed for both areas in DM1^7–9,11,27^; the connection between cortical lobes (*inferior fronto-occipital and longitudinal fasciluli*); and the connection between the limbic system and the frontal lobe (uncinate fasciculus). Regarding the latter, limbic system related difficulties such as emotional recognition have been described in DM1 population^28–30^ and have been found to be age-related^31^. The results reported in our study favor the idea that disrupted networks resulting from WM integrity impairment could be potentially implicated in the various clinical symptoms manifested by the patients. Indeed, previous studies addressing the connectome in DM1 have reported evidence pointing to a relationship between altered networks and clinical symptoms in personality and social cognition, among others^4–7^.

Regarding the progression of WM changes over time, a spatiotemporal pattern of anterio-posterior degeneration is suggested on the basis of our results. This anterio-posterior disconnection gradient has been reported with aging^32–35^, which reinforces the accelerated aging hypothesis in DM1. Notably, the progression reported here appears to start at a very young age (as early as in the 40 s); an observation that is in accordance with the results reported in the most recently published work^14^, which found a reduced FA and increased MD in both cortical and subcortical regions in a group of patients below the age of 40. However, evidence against this neurodegeneration process was reported by Gliem et al. (2019), who found a rather stable pattern of widespread microstructural WM alterations in middle-age patients. The disparities between these results could be partly explained by methodological differences, such as sample size, type of MRI scanner, or the timespan between assessments.

Some authors have suggested that the pattern of degradation in WM integrity could be a mediator of age-related changes in cognitive performance^36^. Neuropsychological performance (and more specifically visuo-construction abilities) was associated with greater WM impairment in this sample, in agreement with the results obtained by Baldanzi et al.^13^. Interestingly, this association was only found at follow-up, when patients differed more from HC in WM damage, suggesting that visuo-construction abilities could be highly sensitive to changes in WM integrity. Both visuospatial and visuo-construction abilities have previously shown to be a hallmark of the DM1 cognitive profile^26,37–39^ and are reliable predictors of both cognitive and structural brain progressive degeneration^14,15^.

Clinical status has also found to be correlated with imaging changes in previous studies, with mixed results^7,11,16,27,40,41^. In the present work, disease severity, as measured by genetic and muscular defect, did not appear to be an indicator of WM impairment. It should be noted that most of the studies that found a correlation with clinical data employed heterogeneous samples including congenital and childhood forms. The present work was restricted to adult and late-onset patients, which could support the notion that, at least in these milder forms of the disease, WM damage might be inherent to the DM1 condition irrespective of disease severity. Nevertheless, we should bear in mind that CTG estimated in peripheral blood has been found to differ from that detected in other tissues (e.g., brain)^42^, which suggests that genotype–phenotype correlations should be interpreted with caution.

The small sample size constitutes the major limitation of this study. The wide timespan in which the sample was assessed increased the rate of experimental attrition, which, along with the inherently higher mortality rate in the DM1 population, resulted in a reduced group of patients for subsequent analyses. For this reason, the results of this study should be interpreted with caution and should not be generalized to the whole DM1 population, given that only the adult and late-onset population has been included. Nonetheless, these findings indicate a potentially greater effect than that observed if the samples were larger. In the future, follow-up studies in other life stages and adding multiple timepoints (>2) with shorter periods between assessments could help to monitor smaller and more specific time-dependent changes and could also help to shed light on whether the natural history of brain changes follows a steady or irregular course. Moreover, future studies should make an effort to standardize MRI protocols, procedural issues, or sample characteristics (e.g., disease form, age span, or time from baseline to follow-up). This would help to provide reliable information on the natural progression of brain changes in DM1, which in turn, would enable the identification of potential biomarkers for the disease for future clinical trials. Finally, other well documented clinical manifestations in DM1 such as fatigue or daytime sleepiness^43^ should also be considered in future studies to obtain a more accurate and comprehensive assessment of brain dysfunction correlates.

Taken together, the current findings suggest that WM degeneration is of progressive nature in DM1, even at early life stages. However, this hypothesis can only be confirmed with further follow-up of the same cohorts. Nevertheless, and precisely because of the young age of the sample, neurodevelopmental changes cannot be fully discarded. This debate can only be fully resolved by brain imaging studies, preferably those of a longitudinal nature that target patients as early as they are diagnosed.

## Conclusion

Adult and late onset DM1 patients might suffer from a slow progressive neurodegenerative process of WM microstructural impairment at early ages, which appears to follow an anterior–posterior gradient and is associated with specific neuropsychological functions (i.e., visuo-construction). The need for further neuroimaging studies assessing patients from a longitudinal approach has been repeatedly highlighted in the literature. The hypothesized neurodegenerative nature of the disease has been supported by age-related changes observed in the brain structure of DM1 patients. However, the scarcity of studies means that the neurodevelopment-neurodegeneration debate still remains open.

## Methods

### Participants

The sample included in this study is part of a longitudinally followed-up cohort. From the 26 DM1 patients and 57 healthy controls (HC) assessed at baseline, 11 DM1 patients from the outpatient Neurology Service of the Donostia University Hospital and 11 HC recruited from healthy volunteers and patients’ relatives were re-scanned. The final sample included for the analyses was composed of 8 DM1 patients and 10 HC. A flow-chart showing the recruitment process and detailing the reasons for each excluded participant is shown in Fig. 3. From the healthy volunteers recruited at baseline, only those whose age, gender and years of education were equal or closely similar (± 5 years at most) to any re-scanned patient were invited to participate in order to form demographically equivalent groups. The DM1 sample was distributed as follows regarding disease onset and inheritance pattern: 4 adult onset (age of onset: 20–40) (50%) and 4 late onset patients (50%) (age of onset > 40); 3 patients with maternal inheritance (37.5%), 4 with paternal inheritance (50%) and 1 inherited from both parents (12.5%).

Patients were excluded from the study if they had congenital (onset at birth), childhood (age of onset: 1–10) or juvenile (age of onset: 10–20) forms, history of major psychiatric or somatic disorder, acquired brain damage or alcohol or drug abuse, presence of corporal paramagnetic body devices that could impede an MRI study, and the presence of cerebral anomalies that could affect the MRI analysis. HC participants were required to satisfy the same inclusion criteria, except for the clinical diagnosis. These criteria were applied both at baseline and at follow-up, the latter referring to the period between both assessments.

This study was reviewed and approved by the Clinical Research Ethics Committee of the Gipuzkoa Health Area (DMRM-2017-01), in accordance with the principles of the Declaration of Helsinki and informed consent was obtained from all participants.

### Clinical and neuropsychological assessment

Clinical and neuropsychological data were obtained at both baseline and follow-up by the research team’s responsible neurologist and neuropsychologist, respectively.

Regarding clinical data, muscular impairment was assessed with the Muscular Impairment Rating Scale (MIRS)^44^, and CTG expansion size was determined from medical registries. Genetic assessment—PCR in DMPK alleles up to approximately 100 CTG and Southern blot analysis for larger expansions—was conducted at both time-points in patients with no recent data available (within the last 5 years). The remaining clinical data concerning clinical form and maternal or paternal inheritance pattern were obtained from the patients’ medical records.

For all DM1 patients, neuropsychological assessment was conducted by an experienced neuropsychologist in the hospital facilities. The examiner was blind to the clinical condition (i.e., disease form, CTG repeats, MIRS or inheritance pattern) and to the MRI results. The administered battery of neuropsychological tests included several tests and subtests, and standardized T values were obtained according to Spanish population-based normative data. The employed assessment tools were: Wechsler Adult Intelligence Scale – Third Edition (WAIS III)^45^, Rey-Osterrieth Complex Figure test (ROCF)^46^, Rey Auditory Verbal Learning Test (RAVLT)^47^, Stroop color and word test^48^, Raven’s Progressive Matrices^49^, verbal fluency (semantic and phonemic)^50^ and California Computerized Assessment Package (CALCAP)^51^.

The converted scores were employed to calculate a mean score representing each of the seven cognitive-domains: visuo-construction (Block design from WAIS-III and ROCF copy), verbal memory (RAVLT immediate recall, RAVLT delayed recall, Total RAVLT), attention/ processing speed (Digit span from WAIS III, STROOP word, STROOP color and the following CALCAP subtests: Simple Reaction Time (RT), election RT, Sequential 1 RT, Sequential 2 RT), executive functioning (Total RAVEN, phonemic fluency, STROOP interference), language (Vocabulary from WAIS-III and semantic fluency), visual memory (ROCF delayed recall), and intellectual functioning. The intellectual functioning domain was estimated from vocabulary and block design subtests of the WAIS III based on Sattler and Ryan (reliability rxx = 0.93; validity r = 0.87).

### MRI acquisition and data preprocessing

Diffusion images were acquired using a 1.5 Tesla scanner (Achieva Nova, Philips) with a single-shot echo-planar diffusion imaging sequence (IVIM-EPI) with the following parameters: voxel size = 1.75 × 1.75 × 2 mm3; 60 axial slices; TR = 9,967 ms; TE = 66 ms; matrix size = 128 × 128. A diffusion gradient was applied across 32 non-collinear directions with b-value = 800 s/mm^2^. Additionally, one set of images was acquired without diffusion weighting (b = 0 s/mm^2^). All participants’ MRI scans were acquired on the same scanner and with the same protocol at both timepoints.

WM tract alterations were assessed with FSL v6.0.4 and Tract-Based Spatial Statistics (TBSS) (Smith et al., 2006) using Fractional Anisotropy (FA) and Mean Diffusivity (MD) images. First, all individual FA/MD images were normalized non-linearly to the HCP1021-2 mm template. Next, the mean image was computed across all participants and skeletonized to obtain the mean FA skeleton, which represents regions with high confidence bundles common to all participants and removing some of the subject-specific tract-based heterogeneities. FA/MD images for each subject were then projected onto the mean skeletons.

WM lesion load was assessed according to the Wahlund scale (ARWM)^53^. When lesions > 5 mm were identified, severity was rated from 0 (no lesions) to 3 (diffuse involvement). Lesion location was quantified separately in five different regions: (1) the frontal area; (2) the parieto-occipital area; (3) the temporal area; (4) the infratentorial area, including the brain stem and cerebellum; and (5) the basal ganglia, including the striatum, globus pallidus, thalamus, internal and external capsules, and insula.

### Statistical analysis

The SPSS (IBM SPSS Statistics 24) statistical package was used for sample description. Data were analyzed through inter-group comparisons to compare DM1 patients and HC, using contingency analysis (Chi-square) for categorical data and parametric (t-test) or non-parametric (Mann–Whitney U) for interval data, when appropriate. Intra-group analysis of the longitudinal evolution of clinical and neuropsychological data was conducted using the Wilcoxon signed-rank test or dependent t-test, when appropriate. In order to control for selective attrition bias, intra-group differences of eligible DM1 patients who were lost to follow-up and those who were included for analyses were conducted through independent sample comparisons (t-test or Mann Whitney U, when appropriate).

For the main objectives of the present work, two analyses were conducted.

### Intergroup and intragroup tract integrity differences: transversal and longitudinal analyses

For diffusion parameters, group comparisons at baseline and at follow-up were conducted using the *randomise* tool included in FSL, a nonparametric permutation test for finding cluster-based significant statistical differences between groups at the voxel level. Global FA and MD values were obtained for each group at both timepoints. Major tracts over which alterations were assessed are shown in Supplementary Fig. 1.

For multiple comparison correction, Threshold-Free Cluster Enhancement (TFCE) (Smith & Nichols, 2009) was used, with 5000 iterations and Family Wise Error (FWE) corrected at *p* = 0.05, thus ensuring that the chance of false positives was no more than 5%, or equivalently, ensuring 95% confidence of no false positives. Group comparisons were conducted using two different contrasts: patient > control, and control > patients.

Global FA and MD (the mean value across all voxels in the brain) and mean values of FA and MD per tract were compared between groups and across longitudinal measures. Similar to previous work^55^, FA and MD group comparisons were assessed following an analysis based on three stages. First, a Shapiro–Wilk statistic was used for testing data normality and justify to which extent parametric or non-parametric statistics should be used. Second, a Kruskal–Wallis test was conducted between all the four possible values (HC baseline, DM1 baseline, HC follow-up, and DM1 follow-up). Finally, a Wilcoxon sum rank test was used for post-hoc analysis for the following four comparisons: DM1 baseline vs HC baseline, DM1 follow-up vs HC follow-up, HC baseline vs HC follow-up, DM1 baseline vs DM1 follow-up. For multiple comparisons across the number of possible tracts, False Discovery Rate (FDR) was applied.

### Association between tract integrity and clinical and neuropsychological data

For assessing the association between skeletonized FA images and both clinical and neuropsychological data, only the intensity values belonging to the significant regions resulting from the group comparison at follow-up were considered. These associations were analyzed at both baseline and at follow-up. For each subject, the mean value of FA within each significant tract was obtained and correlated with CTG expansion size, MIRS score, WM lesion load and the neuropsychological scores using the linear Pearson partial correlation index, controlling for the covariable of age.

In all statistical analyses, missing values were handled by listwise deletion.

## Supplementary Information

Supplementary Information 1.

## Data Availability

The data supporting the findings of this study and analysis code are available from the corresponding author on request.
