# Supplementary material for: White matter integrity changes and neurocognitive functioning in adult-late onset DM1: a follow-up DTI study
Source: Sci Rep. 2022 Mar 7;12:3988. doi: 10.1038/s41598-022-07820-1 (PMC8901711; doi:10.1038/s41598-022-07820-1)
Supplement: Supplementary file 1 — Supplementary Information 1. [file 41598_2022_7820_MOESM1_ESM.pdf]

Supplementary material

White matter integrity changes and neurocognitive functioning in adult-late onset DM1: a follow-up DTI study

Garazi Labayru, Borja Camino, Antonio Jimenez-Marin, Joana Garmendia, Jorge Villanua, Miren Zulaica, Jesus M Cortes, Adolfo López de Munain, and Andone Sistiaga.

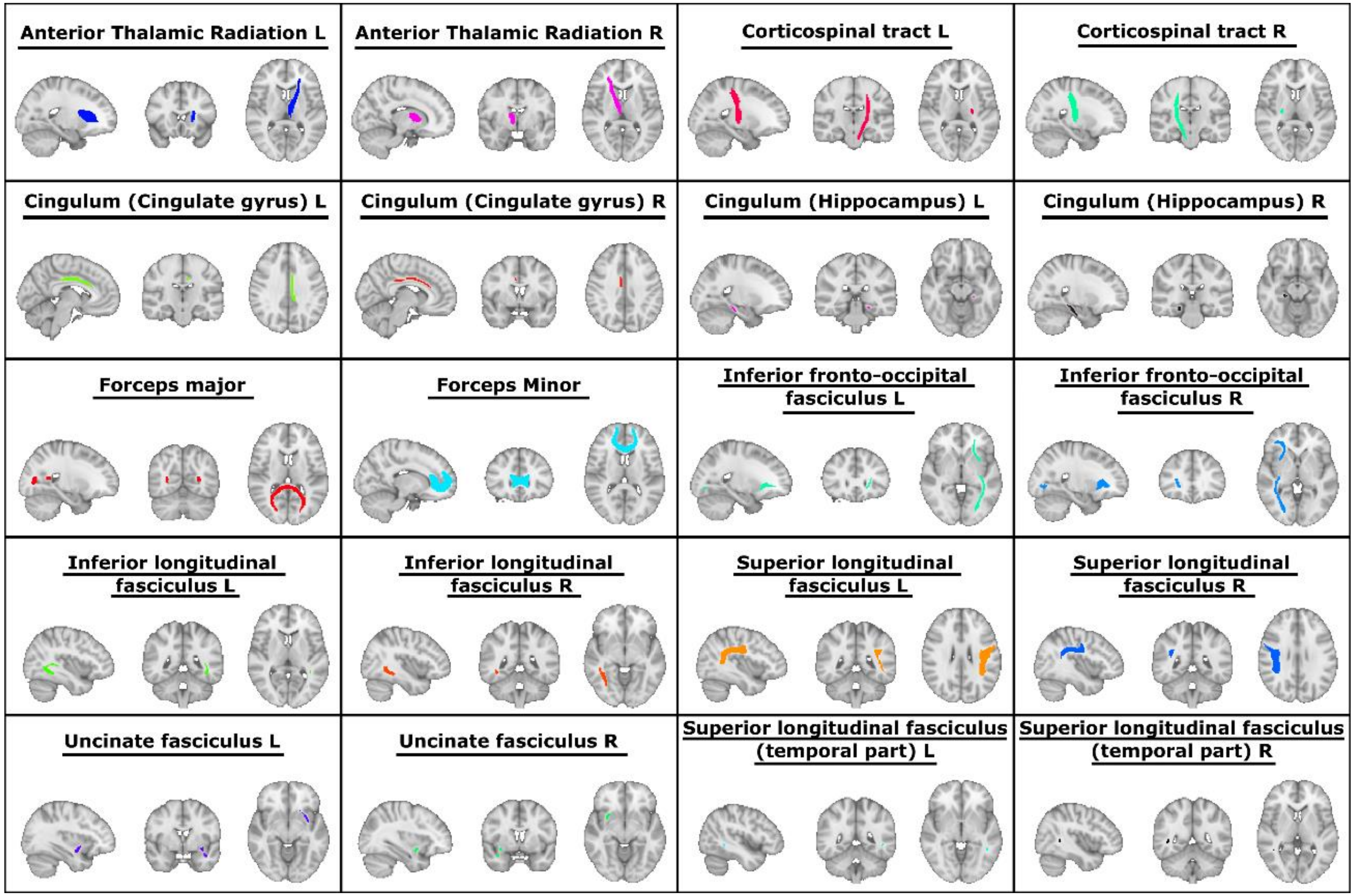

Supplementary Figure 1: Major tracts used for assessing white-matter alterations in DM1.

Supplementary Table 1. Analysis of neuropsychological performance from baseline to follow-up in DM1 patients.

	Baseline		Follow-up				
**Domain**	**Mean**	**(SD)**	**Mean**	**(SD)**	***T***	***p***	***d***
**Attention/Processing speed**	45.13	(5.05)	39.61	(4.26)	2.74	.029	1.37
**Verbal memory**	50.69	(11.14)	45.64	(8.98)	1.15	.289	0.57
**Visual memory**	47.50	(9.75)	46.75	(8.38)	0.19	.857	0.09
**Visuoconstruction**	49.81	(9.45)	47.75	(8.25)	0.69	.513	0.34
**Executive functioning**	45.45	(7.75)	42.06	(9.64)	1.42	.198	0.71
**Language**	48.81	(11.04)	50.88	(3.37	-1.11	.302	.557
**Intellectual Functioning**	97.63	(11.54)	100.00	(10.53)	-0.79	.458	0.39

Note. SD: Standard Deviation

Supplementary Table 2. Group differences in WM integrity FA values.

	Intergroup differences		Post hoc analyses							
			DM1 baseline vs HC baseline		DM1 follow-up vs HC follow-up		HC baseline vs HC follow-up		DM1 baseline vs DM1 follow-up	
	x^2	***p***	***W***	***P***	***W***	***P***	***W***	***p***	***W***	***p***
**Anterior Thalamic Radiation**	192.67	0.000	64	0.034	77	.000	--	--	36	.008
**Corticospinal Tract**	--	--	--	--	71	.004	--	--	33	.039
**Cingulum (Cingulate Gyrus)**	111.60	0.010	--	--	67	.016	47	.049	35	.016
**Cingulum (Hippocampus)**	100.98	0.020	--	--	69	.009	--	--	36	.008
**Forceps Major**	93.07	0.030	--	--	68	.012	--	--	35	.016
**Forceps Minor**	127.54	0.005	--	--	70	.006	--	--	36	.008
**Inferior Fronto-occipital Fasciculus**	18.70	0.000	67	0.016	77	.000	--	--	36	.008
**Inferior Longitudinal Fasciculus**	161.14	0.001	69	0.009	72	.003	--	--	36	.008
**Superior Longitudinal Fasciculus**	174.02	0.001	63	0.043	74	.001	49	.027	36	.008
**Uncinate Fasciculus**	159.39	0.001	73	0.002	69	.009	--	--	--	--
**Superior Longitudinal Fasciculus (Temporal Part)**	95.79	0.020	--	--	--	--	--	--	36	.008
**Global FA**	173.28	0.001	--	--	74	.001	47	.049	36	.008

Note. Only results where statistically significant differences were found are shown (remaining cells are left blank). DM1: Myotonic Dystrophy Type 1; HC: Healthy controls; FA: Fractional Anisotropy. P values > 0.01563 did not survive to FDR multiple-comparison correction.

Supplementary Table 3. Group differences in WM integrity MD values.

	Intergroup differences		Post hoc analyses							
			DM1 baseline vs HC baseline		DM1 follow-up vs HC follow-up		HC baseline vs HC follow-up		DM1 baseline vs DM1 follow-up	
	x^2	***p***	***W***	***P***	***W***	***p***	***W***	***p***	***W***	***p***
**Anterior Thalamic Radiation**	17.2	.001	13	.016	2	.000	48	.037	--	--
**Corticospinal Tract**	8.8	.033	--	--	11	.009	--	--	--	--
**Cingulum (Cingulate Gyrus)**	9.1	.027	17	.043	15	.027	--	--	--	--
**Cingulum (Hippocampus)**	11.8	.008	--	--	16	.034	49	.027	--	--
**Forceps Major**	11.6	.009	--	--	10	.006	49	.027	--	--
**Forceps Minor**	13.5	.004	14	.021	8	.003	--	--	3	.039
**Inferior Fronto-occipital Fasciculus**	22.2	.000	14	.021	1	.000	55	.002	--	--
**Inferior Longitudinal Fasciculus**	21.7	.000	14	.021	1	.000	55	.002	--	--
**Superior Longitudinal Fasciculus**	14.4	.002	14	.021	7	.002	--	--	--	--
**Uncinate Fasciculus**	15.1	.002	12	.012	7	.002	--	--	--	--
**Superior Longitudinal Fasciculus (Temporal Part)**	13.7	.003	12	.012	12	.012	--	--	--	--
**Global MD**	15.4	.002	--	--	5	.001	--	--	--	--

Note. Only results where statistically significant differences were found are shown (remaining cells are left blank). DM1: Myotonic Dystrophy Type 1; HC: Healthy controls; MD: Mean Diffusivity. *P* values > 0.01554 did not survive to FDR multiple-comparison correction.

Supplementary Table 4. Significant regions resulting from group comparisons (DM1 < HC) at baseline.

Tract (JHU ICBM)	Peak X (MNI)	Peak Y (MNI)	Peak Z (MNI)	Peak p	Peak t	Mean t	N Voxels	Vol (mm^3)
**Inferior Fronto-occipital Fasciculus R**	28	16	-4	.044	4.8354	2.9123	20	28
**Inferior Longitudinal Fasciculus R**	42	-10	-20	.046	4.6402	3.249	14	22
**Uncinate Fasciculus R**	32	2	-10	.049	3.5133	2.6954	10	18

Note. Only tracts with N voxels greater than 10 are shown. L: left; R: right

Supplementary Table 5. Significant regions resulting from group comparisons (DM1 < HC) at follow-up.

Tract	Peak X (MNI)	Peak Y (MNI)	Peak Z (MNI)	Peak p	Peak t	Mean t	N Voxels	Vol (mm^3)
**Anterior Thalamic Radiation L**	-10	0	-2	.003	4.3372	1.9391	63	504
**Anterior Thalamic Radiation R**	18	18	0	.002	3.979	1.89	51	408
**Corticospinal Tract L**	-24	-16	6	.004	3.1946	1.7549	46	368
**Corticospinal Tract R**	22	-16	-2	.002	3.1426	1.9909	47	376
**Cingulum (Cingulate Gyrus) L**	-8	-24	32	.004	3.04	2.0445	15	120
**Cingulum (Hippocampus) R**	24	-24	-18	.008	4.0946	2.0253	25	200
**Forceps Major**	22	-80	8	.043	3.5965	1.8643	135	1080
**Forceps Minor**	-14	44	-10	.002	5.9171	2.1132	528	4224
**Inferior Fronto-occipital Fasciculus L**	-34	2	-6	.002	5.7826	2.0435	202	1616
**Inferior Fronto-occipital Fasciculus R**	40	-22	-10	.002	4.7534	2.0815	243	1944
**Inferior Longitudinal Fasciculus L**	-46	0	-16	.002	5.3999	2.183	142	1136
**Inferior Longitudinal Fasciculus R**	44	-8	-14	.005	5.2797	2.3102	82	656
**Superior Longitudinal Fasciculus L**	-38	-44	16	.002	4.9712	1.9591	237	1896
**Superior Longitudinal Fasciculus R**	36	-10	24	.004	4.6874	2.1655	195	1560
**Uncinate Fasciculus L**	-40	4	-26	.002	2.907	1.7682	38	304

Note. Only tracts with N voxels greater than 10 are shown. L: left; R: right.

Supplementary Table 6. Correlation analyses (Pearson r) between clinical and neuropsychological data and regional and global FA at follow-up.

Domain	Anterior Thalamic Radiation	Corticospinal Tract	Cingulum (Cingulate Gyrus)	Cingulum (Hippocampus)	Forceps Major	Forceps Minor	Inferior Fronto-occipital Fasciculus	Inferior Longitudinal Fasciculus	Superior Longitudinal Fasciculus	Uncinate Fasciculus	Superior Longitudinal Fasciculus (Temporal Part)	Global FA
**Attention/ Processing speed**	-0.33	-0.059	0.02	0.146	-0.44	-0.022	-0.33	-0.346	-0.182	-0.615	-0.206	-0.252
**Verbal memory**	-0.26	0.062	0.277	0.35	0.112	0.005	-0.126	-0.09	-0.22	-0.146	0.153	0.075
**Visual memory**	-0.213	0.506	0.341	0.154	0.205	-0.051	-0.076	0.107	0.157	0.191	0.029	0.269
**Visuo-construction**	0.733	0.581	0.663	0.692	0.772*	0.778*	0.798*	0.7	0.614	0.739	0.695	0.788*
**Executive functioning**	-0.638	-0.138	0.109	-0.017	-0.367	-0.252	-0.555	-0.519	-0.418	-0.564	-0.19	-0.382
**Intellectual Functioning**	0.032	0.45	0.839*	0.511	0.543	0.472	0.245	0.213	0.228	0.387	0.614	0.37
**CTG**	-0.511	-0.239	-0.18	-0.463	-0.651	-0.461	-0.705	-0.727	-0.479	-0.581	-0.67	-0.52
**MIRS**	-0.34	-0.475	-0.337	-0.744	-0.442	-0.442	-0.476	-0.533	-0.368	-0.237	-0.41	-0.618
**WM lesion load**	-0.64	-0.64	-0.28	-0.56	-0.64	-0.57	-0.79*	-0.88**	-0.8*	-0.66	-0.55	-0.76*

Note. * *p*<0.05; ** *p*<0.01. FA: Fractional Anisotropy;
